# Scarless gene disruption enabled by a dual-plasmid knockout platform in a clinical infant-derived *Bifidobacterium breve* strain

**DOI:** 10.3389/fmicb.2025.1653505

**Published:** 2025-09-10

**Authors:** Zhenxuan Gao, Lihui Feng

**Affiliations:** Institutes of Biomedical Sciences, Fudan University, Shanghai, China

**Keywords:** *Bifidobacterium breve*, infant gut microbiota, probiotic strain engineering, dual-plasmid knockout system, scarless genome editing

## Abstract

In the developing gut of infants, *Bifidobacteria* establish themselves and become one of the predominant microbial populations, playing vital roles in host health by modulating immune responses, inhibiting the growth of pathogenic bacteria, and enhancing nutrient metabolism. While *Bifidobacterium* strains from Western populations have been extensively studied, those derived from Chinese infants remain underexplored. Given the substantial impact of geography, diet, and host genetics on gut microbiota composition and function, strains from the Chinese population may possess unique probiotic properties with significant scientific and clinical relevance. In this study, we isolated a highly abundant clinical *Bifidobacterium breve* strain with intrinsically high transformation efficiency from the feces of a healthy Chinese infant. We obtained its complete genome using Oxford Nanopore sequencing. To assess its genetic tractability, we first employed two conventional double-crossover gene knockout strategies. A *pyrE* mutant was successfully constructed using a shuttle vector, leveraging its 5-fluoroorotic acid (5-FOA) sensitivity as a counterselection marker. To enable efficient, scarless genome editing, we developed a novel dual-plasmid system that markedly improved the selection of single-crossover events. This approach enabled robust and flexible genetic manipulation of a clinically derived *B. breve* strain that was previously recalcitrant to standard knockout techniques. Our work not only provides a powerful platform for dissecting the probiotic mechanisms of *B. breve*, but also serves as a valuable reference for the development of genetic tools applicable to other clinically relevant strains.

## 1 Introduction

*Bifidobacterium* is a key member of the human gut microbiota, contributing to nutrient metabolism, immune regulation, and the maintenance of intestinal barrier integrity (O’Callaghan and van Sinderen, 2016; Sun et al., 2020; Gavzy et al., 2023; Segui-Perez et al., 2025). In early infancy, *Bifidobacterium* typically dominates the gut microbial community. Studies have shown that in healthy, breastfed infants aged 1 week to 6 months, *Bifidobacterium* can constitute up to 90% of the total microbiota in some individuals (Arrieta et al., 2014; Arboleya et al., 2016; Hidalgo-Cantabrana et al., 2017; Hill et al., 2017). This dominance is largely due to the ability of infant-adapted species, such as *Bifidobacterium bifidum*, *Bifidobacterium breve*, *Bifidobacterium longum* subsp. *infantis*, and *Bifidobacterium longum* subsp. *longum*—to efficiently metabolize human milk oligosaccharides (HMOs), granting them a competitive edge in the infant gut (Ojima et al., 2022). These species not only promote nutrient absorption and immune development in infancy (Gensollen et al., 2016; Henrick et al., 2021; Lin et al., 2022), but also exert long-term effects on host metabolic, immune, and neurological health by modulating the gut–brain axis through bioactive metabolites like short-chain fatty acids and indole derivatives (Kobayashi et al., 2017; Cheng et al., 2024; Qian et al., 2024; Ragavan and Hemalatha, 2024).

As infants transition to solid foods during weaning, the nutrient landscape of the gut shifts dramatically. Correspondingly, the relative abundance of *Bifidobacterium* declines to 2–14% (Arboleya et al., 2016), and the community composition evolves from infant-type species to more adult-associated taxa such as *Bifidobacterium adolescentis*, *Bifidobacterium pseudocatenulatum*, *Bifidobacterium catenulatum*, and *B. longum* subsp. *longum* (Kato et al., 2017; Xiao et al., 2024). This succession reflects not only dietary changes but also the adaptive plasticity of the gut microbiota in response to host development.

Despite the recognized importance of *Bifidobacterium* in health and disease, most existing studies remain correlational due to the technical challenges of performing mechanistic analyses, especially in clinical isolates. Genetic manipulation in *Bifidobacterium* is hindered by low transformation efficiency, largely attributable to their strict anaerobic growth requirements and active restriction–modification (R–M) systems, which substantially limit the application of molecular tools (Brancaccio et al., 2013; Zuo and Marcotte, 2021).

Traditional genetic methods in *Bifidobacterium* primarily rely on homologous recombination. Single-crossover insertional mutagenesis is frequently used due to its simplicity; however, it heavily depends on transformation efficiency and suffers from issues such as mutation instability, polar effects on downstream genes, and the permanent integration of antibiotic resistance markers (Hirayama et al., 2012). To date, this approach has been successfully implemented in only a few strains, such as *B. breve* UCC2003 (Pokusaeva et al., 2010; Pokusaeva et al., 2011; Christiaen et al., 2014; Egan et al., 2014a; Egan et al., 2014b; James et al., 2018; Kelly et al., 2020) and *B. longum* subsp. *infantis* ATCC 15697 (JCM 1222) (Sakanaka et al., 2019; Hirano et al., 2021; Arzamasov et al., 2022). In contrast, double-crossover allelic exchange strategies using suicide plasmids offer more stable and cleaner gene knockouts; however, their implementation requires even higher transformation efficiencies and still results in the insertion of a selection marker (Higgins and Ryan, 2021).

To overcome these limitations, scarless gene deletion techniques based on two-step homologous recombination using shuttle plasmids have been developed. These methods allow precise, marker-free genome editing and are less dependent on transformation efficiency. However, they often require multiple recombination steps and intensive screening. Efforts to improve the efficiency of the first homologous recombination step have led to the development of temperature-sensitive (Ts) plasmids (Sakaguchi et al., 2012; Kozakai et al., 2024) and inducible plasmid self-destruction (IPSD) systems (Zuo et al., 2019). Additionally, plasmid incompatibility-based approaches have been used to facilitate the second recombination event (Hirayama et al., 2012). Nonetheless, the lack of reliable counterselection systems remains a major bottleneck for the efficient isolation of desired mutants in *Bifidobacterium*.

Recent advances include the application of CRISPR-Cas systems (Han et al., 2022; Pan et al., 2022; Li et al., 2023; Han et al., 2024; Lin et al., 2025) and the construction of transposon mutant libraries (Ruiz et al., 2013; Sakanaka et al., 2018; Shiver et al., 2025), which have shown promise for genome-wide functional studies. However, these tools remain limited in scope due to strain-specific barriers and persistently low transformation efficiencies, especially in clinical isolates.

Importantly, most *Bifidobacterium* strains with established genetic tools originate from Western or Japanese sources, such as *B. breve* UCC2003 (Ireland), *B. longum* subsp. *longum* NCC2705 (Switzerland), *B. longum* subsp. *infantis* ATCC 15697 and *B. bifidum* ATCC 15696 (United States), and *B. longum* subsp. *longum* 105-A and *B. bifidum* PRL2010 (Japan). In contrast, genetically tractable strains from the Chinese population, especially infants, have not been systematically investigated. Given the well-documented differences in gut microbial composition associated with geography, ethnicity, diet, and host genetics (Senghor et al., 2018), *Bifidobacterium* strains from Chinese infants may possess unique physiological traits suited to their local environments and populations. Systematic isolation and characterization of such strains is therefore essential not only for understanding their biology but also for the development of locally relevant probiotic interventions and genetic tools.

In this study, we isolated a clinically relevant *B. breve* strain, designated *B. breve* GZX43, from the feces of a healthy Chinese infant. This strain exhibited naturally high transformation efficiency. While current genetic manipulation approaches based on suicide plasmids proved ineffective in this clinical isolate due to strain-specific barriers, we constructed a *pyrE* mutant using a shuttle vector via the counterselectable phenotype of 5-fluoroorotic acid (5-FOA) sensitivity. Furthermore, we developed and utilized a novel dual-plasmid system for efficient, scarless genome editing. Adopting this approach, we generated knockout mutants of GE001229 in *B. breve* GZX43. Our system provides a flexible and robust toolkit for genome engineering in clinically relevant *Bifidobacterium* strains. This work establishes a strong technical foundation for future studies of functional genomics, host–microbe interactions, and the rational development of precision probiotics tailored to Chinese infant populations.

## 2 Materials and methods

### 2.1 Fecal sample collection

Fecal samples were obtained from three healthy, full-term infants who were vaginally delivered and exclusively breastfed at 28 days of age in Shanghai, China (Table 1). The study was approved by the Ethics Committee of the Children’s Hospital of Fudan University (Reference No. 012 (2019)). Samples were collected in sterile containers, promptly frozen at −80°C, and stored under these conditions until further processing.

**Table 1:** Fecal sample collection and donor information.

Sample ID	Donor sex	Gestational age (d)	Mode of delivery	Feeding method	Sampling age (d)
S1	Male	274	Vaginal delivery	Breastfeeding	28
S2	Male	276	Vaginal delivery	Breastfeeding	28
S3	Male	271	Vaginal delivery	Breastfeeding	28

### 2.2 Metagenomic sequencing and analysis

Metagenomic sequencing and bioinformatic analysis were conducted by OE Biotech Co., Ltd. (Shanghai, China). Total DNA was extracted using a QIAamp® Fast DNA Stool Mini Kit (Qiagen). DNA was quantified via NanoDrop 2000 (Thermo Fisher) and qualified via agarose gel electrophoresis. DNA was fragmented with FEA Enzyme Mix (TIANGEN) and purified using Agencourt AMPure XP beads (Beckman Coulter).

Sequencing libraries were prepared with a TruSeq Nano DNA LT Sample Preparation Kit (Illumina) and sequenced on an Illumina NovaSeq 6,000 platform to produce 150 bp paired-end reads. Raw FASTQ sequences were quality-trimmed using fastp (v0.20.1). Host-derived reads were removed by aligning to the human reference genome with bbmap (v38.93–0); aligned reads were discarded. Remaining high-quality reads were assembled *de novo* using MEGAHIT (v1.2.9).

Open reading frames (ORFs) were predicted from contigs >500 bp using prodigal (v2.6.3) and translated into amino acid sequences. Non-redundant gene sets were clustered with MMSeqs2 (v13.45111) at 95% sequence identity and 90% coverage; the longest gene per cluster was selected as the representative sequence. Clean reads from each sample were aligned to the non-redundant gene set (95% identity threshold) using salmon (v1.8.0) to calculate gene abundance.

Representative gene sequences were annotated against the NCBI Nr database using DIAMOND (v2.1.9) with an E-value cutoff of < 1e−5. Based on the annotation, gene abundances were aggregated to identify taxonomic profiles across multiple levels: Kingdom, Phylum, Class, Order, Family, Genus, and Species. Species-level relative abundances across three fecal samples are presented in Supplementary Table S1. At the species level, bar plots were generated to display the top 15 most abundant species. A heatmap of the top 30 species was constructed to illustrate distribution across samples, with hierarchical clustering used to identify dominant taxa and assess microbial community structure. A phylogenetic tree was generated to visualize community composition, and a Venn diagram was used to identify shared and unique species among the groups. All visualizations were created using R (v4.1.2).

### 2.3 Isolation and identification of *Bifidobacterium*

Each fresh fecal sample (40 mg) was suspended in 1 mL of reduced phosphate-buffered saline (PBS) supplemented with 0.05% L-cysteine-HCl. Samples were vortexed in five 30-s cycles with 30-s intervals to fully homogenize fecal matter. Homogenates were transferred to an anaerobic Coy chamber (atmosphere: 75% N_2_, 20% CO_2_, 5% H_2_). An equal volume of cryopreservation medium (30% [v/v] glycerol, 70% [v/v] PBS, and 0.5 g/L L-cysteine-HCl) was added. After thorough mixing, suspensions were aliquoted into 2 mL glass crimp-top vials, sealed, and stored at −80°C.

For bacterial isolation, aliquots were serially diluted in reduced PBS to 10^−1^–10^−5^. The 10^−5^ dilution was plated on LYHBHI agar (37 g/L BHI, 5 g/L yeast extract, 1 g/L cellobiose, 1 g/L maltose, 0.5 g/L L-cysteine-HCl, 5 mg/L hemin, 15 g/L agar) and incubated anaerobically at 37°C for 48–72 h. From each sample, 40 colonies were randomly selected and inoculated into LYHBHI broth in 96 deep-well plates (Nest). Plates were sealed with gas-permeable membranes (Sigma) and incubated anaerobically at 37°C for 48–72 h. Subsequently, 100 μL of each culture was transferred into 96 shallow-well plates (Nest) containing 100 μL of cryopreservation medium. These plates were sealed with aluminum foil and stored at −80°C.

Genomic DNA was extracted from cultures in the 96 deep-well plates following a previously described protocol (Zhou and Feng, 2024). Full-length 16S rRNA genes were amplified using primers 27-F (5’-AGAGTTTGATCCTGGCTCAG-3′) and 1,492-R (5’-TACGGYTACCTTGTTACGACTT-3′) for taxonomic identification (primers No. 1–2).

### 2.4 Whole-genome sequencing and assessment

Genomic DNA from the *B. breve* clinical isolate GZX43 was subjected to whole-genome sequencing using the Oxford Nanopore Technologies MinION platform (Biomarker Technologies Co., Ltd., Beijing, China). Raw data were generated in binary fast5 format, containing complete signal traces, with each read in an individual file. Base calling was performed using Guppy (v3.2.6) within the MinKNOW suite to convert fast5 files into FASTQ format for downstream analysis.

Raw reads were filtered to remove low-quality reads, adapter sequences, and reads shorter than 2,000 bp, yielding high-quality data for assembly. Assembly was performed with Canu (v1.5), followed by genome circularization using Circlator (v1.5.5) to produce complete circular genomes.

Coding sequences (CDSs) were predicted with prodigal (v2.6.3). Transfer RNA (tRNA) genes were identified using tRNAscan-SE (v2.0), while ribosomal RNA (rRNA) and non-coding RNAs (ncRNAs) were detected using Infernal (v1.1.3). Prophage regions were identified with PhiSpy (v2.3), and horizontal gene transfer (HGT) events were predicted using IslandPath-DIMOB (v0.2). CRISPR arrays were detected with CRT (v1.2). Functional annotation of predicted proteins was performed by aligning against the eggNOG database using BLAST, with an E-value threshold of 1e−5.

### 2.5 Construction and visualization of the phylogenetic tree

A phylogenetic tree was constructed based on 16S rRNA gene sequences of *Bifidobacterium* species using MEGA X (v10.2.6) with the maximum likelihood method. Tree annotation and visualization were performed using iTOL (v6). Whole-genome sequencing and analysis of the studied *Bifidobacterium* strains were conducted by Biomarker Technologies Co., Ltd. (Beijing, China) using Oxford Nanopore Technologies platforms. Genomic features—including genome size, CDSs, HGT elements, prophage regions, and CRISPR-Cas systems—were extracted from the sequencing data and mapped onto the phylogenetic tree. These features were color-coded to enable intuitive visualization.

### 2.6 Bacterial strains, media, and culture conditions

All bacterial strains used in this study are listed in Supplementary Table S2. Five *Bifidobacterium* strains—*B. dentium* LFYP24, *B. pseudocatenulatum* LFYP29, *B. adolescentis* LFYP80, *B. breve* LFYP81, and *B. longum* subsp. *longum* LFYP82—were previously isolated from the gut microbiota of a healthy infant in the United States (Feng et al., 2020). *B. bifidum* JCM 1254 and *B. longum* subsp. *infantis* ATCC 15697 were obtained from the China Center of Industrial Culture Collection (CICC). *B. breve* GZX43 was isolated in this study from the fecal sample (S3) of a healthy infant in China.

*Bifidobacterium* strains were cultured anaerobically in LYHBHI broth at 37°C using an anaerobic Coy chamber. *Escherichia coli* DH5α, used as the host strain for plasmid propagation, was grown aerobically in LB medium at 37°C. For solid media, 15 g/L agar was added before autoclaving. When needed, selective agents were incorporated as follows: spectinomycin (Sp) at 100 μg/mL for both *E. coli* and *Bifidobacterium*; chloramphenicol (Cm) at 20 μg/mL for *E. coli* and 5 μg/mL for *Bifidobacterium*. 5-Fluoroorotic acid (5-FOA) was used at 1000 μg/mL for counterselection and at 100–5000 μg/mL to assess the 5-FOA resistance of *B. breve* GZX43 wild-type and Δ*pyrE* strains. Bifidobacterial minimal medium (BMM) was prepared as previously described (Sakaguchi et al., 2013), supplemented with 1.8 mM UMP and uracil. Bacterial growth was monitored by measuring optical density at 600 nm (OD_600_) using a GENESYS 30 spectrophotometer (Thermo Fisher).

### 2.7 DNA manipulation and plasmid construction

Primers, template DNAs, and plasmids used are listed in Supplementary Table S3. Primers were designed using SnapGene v4.1.8^1^ and synthesized by Sangon Biotech (Shanghai, China). High-fidelity PCR was performed using KOD-Plus-Neo DNA polymerase (TOYOBO) with purified genomic DNA as the template. For 16S rRNA-based strain identification, Premix Taq (Takara) was used. Genomic DNA was extracted following established protocols (Zhou and Feng, 2024), and plasmid DNA was isolated using a Plasmid DNA Mini Kit I (Omega).

The amplified fragments were isolated via 1% (w/v) agarose gel electrophoresis. DNA fragments for plasmid assembly were extracted using a QIAquick Gel Extraction Kit (Qiagen). All plasmids were constructed via one-step cloning using a ClonExpress II One Step Cloning Kit (Vazyme). Sanger sequencing was performed by Map Biotech (Shanghai, China).

Electroporation-ready plasmids were constructed by one-step assembly. In pElectro-4.3 kb, the *Bifidobacterium* and *E. coli* replicons and the spectinomycin resistance (Sp^r^) gene were amplified from the shuttle vector pMDY23 (derived from pDP870; primers No. 3–6). To evaluate plasmid size effects on transformation efficiency, three larger constructs—pElectro-5.2 kb (primers No. 7–10), pElectro-6.6 kb (No. 11–18), and pElectro-7.6 kb (No. 19–26)—were generated. These shared the same backbone and Sp^r^ marker as pElectro-4.3 kb but carried non-coding genomic inserts of 900, 2,300, and 3,300 bp, respectively, from intergenic regions of *B. breve* GZX43.

Suicide vectors pSUC43-ΔGE000081 and pSUC43-ΔGE001410 were constructed by amplifying ~500 bp regions upstream and downstream of the target genes from *B. breve* GZX43 genomic DNA. These fragments were assembled with a non-replicative plasmid backbone containing the *E. coli* replicon and Sp^r^ gene from pMDY23 (primers No. 27–34 for pSUC43-ΔGE000081 and No. 35–42 for pSUC43-ΔGE001410). The Sp^r^ cassette was placed between the homologous arms.

To construct the shuttle plasmid pKO43-Δ*pyrE*, 552 bp upstream (primers No. 47–48) and 508 bp downstream (No. 49–50) of the *pyrE* gene were PCR-amplified from *B. breve* GZX43. As with other constructs, the *Bifidobacterium* and *E. coli* replicons and the Sp^r^ gene were derived from pMDY23 (primers No. 43–46). The Sp^r^ cassette was placed outside the homologous arms to allow markerless *pyrE* deletion via double-crossover recombination.

For targeted deletion of GE001229, pALox-ΔGE001229 was assembled using 614 bp upstream (primers No. 57–58) and 521 bp downstream (No. 59–60) sequences. The *Bifidobacterium* replicons, together with the *E. coli* replicons and Sp^r^ gene, were amplified from the shuttle plasmid pMDY23 (primers No. 51–56). The final construct featured the upstream and downstream homology arms along with the Sp^r^ cassette, all flanked by directly oriented lox66 and lox71 sites. These lox sites were introduced by incorporating their sequences into the primer overhangs (primers No. 51 and 55). All fragments were assembled by seamless one-step cloning.

To construct the Cre recombinase-expressing helper plasmid pBCre, the *cre* gene from Enterobacteria phage P1 (primers No. 65–66) was placed under the control of the strong promoter pBL1363, PCR-amplified from *B. longum* subsp. *longum* NCC2705 genomic DNA (primers No. 63–64). The chloramphenicol resistance gene (*cat*) was amplified from plasmid pLF103 (primers No. 69–70). The expression cassette, *cat*, and shuttle backbone containing *Bifidobacterium*/*E. coli* replicons (primers No. 61–62 and 67–68) were assembled via seamless cloning.

### 2.8 Transformation of *Bifidobacterium*

*Bifidobacterium* strains were activated by overnight culture in LYHBHI broth and diluted to an OD_600_ of 1.0. Cultures were then inoculated 1:50 into fresh LYHBHI (50 mL total) and grown for 4–6 h until OD_600_ reached 0.4–0.6. All subsequent steps were performed on ice. Cells were harvested by centrifugation (4,000 rpm, 4°C, 10 min), washed twice with ice-cold sucrose-citrate buffer (0.5 M sucrose, 1 mM citrate, pH 6.0), and resuspended in the same buffer. To normalize transformation efficiency across strains, the resuspension volume was adjusted so that each 50 μL aliquot contained exactly 2.5 OD_600_ units.

For electroporation, 50 μL of competent cells were mixed with 1 μg of plasmid DNA and incubated on ice for 10 min. Electroporation was performed in 0.2 cm cuvettes using a Gene Pulser Xcell (Bio-Rad) with settings of 2.4 kV/cm, 25 μF, and 200 *Ω*. Immediately after the pulse, 1 mL of LYHBHI was added, and the culture was incubated anaerobically at 37°C for 3 h. Cells were then plated on selective LYHBHI agar containing 100 μg/mL Sp or 5 μg/mL Cm and incubated anaerobically at 37°C for 3–4 days.

Transformation variability under identical conditions was evaluated by introducing pElectro-4.3 kb into eight *Bifidobacterium* strains. To assess the impact of plasmid size on transformation efficiency, *B. breve* GZX43 was transformed with pElectro-4.3 kb, pElectro-5.2 kb, pElectro-6.6 kb, and pElectro-7.6 kb. Transformation efficiency was quantified by counting colony-forming units (CFU) on Sp-supplemented plates post-electroporation.

### 2.9 Gene knockout via suicide vector

Targeted deletions of GE000081 and GE001410 in *B. breve* GZX43 were achieved using a double-crossover allelic exchange approach. The suicide plasmids pSUC43-ΔGE000081 and pSUC43-ΔGE001410 were independently introduced into *B. breve* GZX43 via electroporation. Following anaerobic incubation at 37°C for 5 days, transformants were selected on Sp-supplemented media. Genomic DNA from individual colonies was extracted, and PCR was performed using the primer pairs GE000081 left-F/right-R (primers No. 71–72) and GE001410 left-F/right-R (No. 73–74) to distinguish single-crossover (SCO) and double-crossover (DCO) recombinants from wild-type (WT) colonies.

### 2.10 Knockout of *pyrE*

To delete *pyrE*, *B. breve* GZX43 cells were electroporated with pKO43-Δ*pyrE* and plated on LYHBHI agar supplemented with Sp. Resulting transformants were cultured in antibiotic-free LYHBHI broth to promote DCO recombination. Cultures were then plated onto LYHBHI agar containing 1,000 μg/mL 5-FOA and incubated anaerobically at 37°C for 48 h. Single colonies exhibiting resistance to 5-FOA were isolated and further cultured in LYHBHI broth supplemented with 5-FOA. After overnight incubation, visible turbidity indicated successful growth. Genomic DNA was extracted, and PCR followed by Sanger sequencing using primers *pyrE* left-F and right-R (primers No. 75–76) confirmed deletion of the *pyrE* gene.

Phenotypic characterization of Δ*pyrE* strains included resistance and growth assays. For resistance assays, WT and Δ*pyrE* strains were grown overnight, adjusted to an OD_600_ of 1.0, and diluted 1:1000 into fresh LYHBHI medium containing 5-FOA at final concentrations of 0, 100, 300, 500, 700, 1,000, 1,500, 2,000, 3,000, or 5,000 μg/mL. Cultures were incubated anaerobically at 37°C for 48 h before OD_600_ measurements. For growth assays under varying nutritional conditions, pre-cultured WT and Δ*pyrE* strains (OD_600_ = 1.0) were diluted and plated on LYHBHI, BMM, BMM supplemented with 1.8 mM UMP, and BMM supplemented with 1.8 mM uracil. All conditions were tested in triplicate. Plates underwent anaerobic incubation at 37°C for 72 h, followed by colony-forming units (CFU) enumeration.

### 2.11 Knockout of GE001229

The knockout plasmid pALox-ΔGE001229, carrying homologous arms, was introduced into *B. breve* GZX43 by electroporation. Transformants were selected on LYHBHI agar containing Sp. A confirmed transformant was rendered competent and electroporated with the helper plasmid pBCre, followed by selection on LYHBHI agar containing both Sp and Cm. Single colonies were cultured in liquid LYHBHI supplemented with both antibiotics. Genomic DNA was extracted, and PCR was performed using primer pairs pALox-ΔGE001229-Scr-F/R (primers No. 77–78) and pBcre-Scr-F/R (No. 79–80) to confirm the presence of both plasmids.

To promote the loss of intact pALox-ΔGE001229, dual-plasmid transformants were subcultured for three passages in LYHBHI broth containing only Cm (OD_600_ = 1.0, diluted 1:1000 into fresh medium per passage, 24-h anaerobic incubation). Cultures were plated on LYHBHI agar with both antibiotics to select for SCO recombinants. SCO candidates were identified by PCR using GE001229 left-F and right-R primers (primers No. 81–82).

For DCO mutant generation, a confirmed SCO colony was subcultured twice in Sp-free LYHBHI (OD_600_ = 1.0, 1:1000 dilution, 24 h anaerobic incubation). Cultures underwent serial dilution and were plated onto antibiotic-free LYHBHI agar. Ninety-six colonies were randomly picked and replica-inoculated into 200 μL LYHBHI with or without Sp, followed by 24 h anaerobic incubation. Sp-sensitive colonies were identified and verified by PCR using GE001229 left-F and right-R primers (primers No. 81–82). Successful gene deletion was confirmed via Sanger sequencing.

Loss of plasmids in SCO and DCO mutants was assessed by PCR. For plasmid pALox, primer pair pALoxCheck-1-F/R (primers No. 83–84) amplified the lox71 region from the intact plasmid, while pALoxCheck-2-F/R (primers No. 85–86) amplified the integrated Sp^r^-homology cassette. For plasmid pBCre, primer pair pBCre-Scr-F/R (primers No. 79–80) amplified the Cm^r^ region. To evaluate the stability of pBCre, DCO mutants were subcultured four times in antibiotic-free LYHBHI medium (OD_600_ = 1.0, diluted 1:1000 into fresh medium per passage, 24-h anaerobic incubation), then replica-plated onto Cm-containing and antibiotic-free agar. PCR verification was performed using pooled cultures from each passage and Cm-sensitive single colonies isolated from the fourth subculture.

### 2.12 Quantification and statistical analysis

Statistical analyses were conducted using GraphPad Prism 9.0.^2^ Data are presented as mean ± standard error of the mean (SEM). One-way ANOVA followed by Tukey’s multiple comparisons test was used for multi-group comparisons. Statistical significance was defined as follows: not significant (ns), *p* > 0.05; ****p < 0.0001.

### 2.13 Reagents used for this research

All reagents used in this work are presented in Supplementary Table S4.

## 3 Results

### 3.1 Recovery and profiling of *Bifidobacterium* strains from healthy Chinese infants

**Figure 1:**
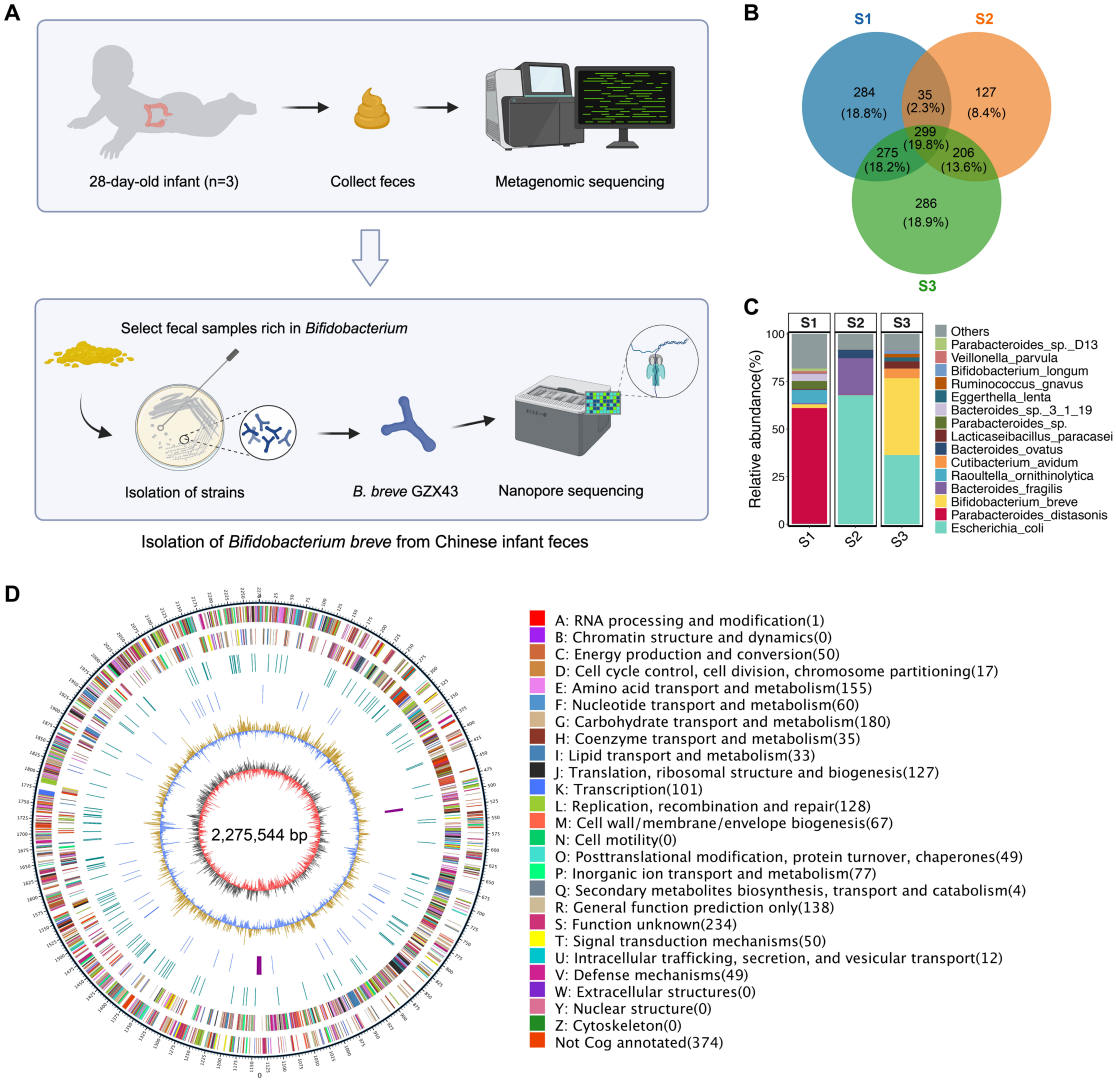
Isolation and genomic characterization of a *B. breve* strain from feces of healthy Chinese infants. **(A)** Workflow of strain isolation. Metagenomic sequencing was performed on fecal samples from three 28-day-old healthy Chinese infants. The sample with the highest *Bifidobacterium* abundance (S3) was selected for bacterial isolation, yielding *B. breve* strain GZX43, which was subsequently sequenced using Nanopore technology. **(B)** Venn diagram showing species-level microbial differences among the three fecal samples (see Table 1 for details). **(C)** Relative abundance of the top 15 microbial species in each fecal sample, based on metagenomic profiling. **(D)** Circos plot of the complete genome of *B. breve* GZX43 obtained via Nanopore sequencing. The outermost ring indicates genomic coordinates with 5 kb tick marks. The second and third rings show forward and reverse strand protein-coding genes, color-coded by COG functional categories. The fourth ring displays repetitive sequences; the fifth ring shows non-coding RNAs (tRNAs in blue, rRNAs in purple). The sixth ring represents GC content, with yellow denoting regions above the average and blue indicating below-average values. The innermost ring depicts GC skew (gray for G > C, red for C > G).

Most *Bifidobacterium* strains characterized to date have been isolated from Western populations (Feng et al., 2019; Taft et al., 2022; Güler et al., 2024), where gut microbiota composition and dietary habits differ substantially from those in China. Isolating indigenous *Bifidobacterium* strains from Chinese infants not only addresses a significant regional knowledge gap but also enhances the applicability of probiotic research to local health contexts. Figure 1A illustrates the workflow for isolating *Bifidobacterium* from fecal samples of healthy Chinese infants.

We collected stool samples from three full-term, vaginally delivered, breastfed 28-day-old infants born in Shanghai, China (Table 1), and performed metagenomic sequencing to profile their gut microbiota. Despite similar age, delivery mode, feeding status, and geographic origin, the microbiota compositions of the three infants (S1, S2, and S3) exhibited substantial inter-individual variability. Only 19.8% of species-level taxa were shared across all three samples, underscoring the high heterogeneity in early-life gut microbial communities (Figure 1B). Bacterial communities exhibited higher relative abundance and greater divergence than archaeal or viral populations (Supplementary Figure S1A). Species-level abundance profiles further highlighted distinct microbial architectures: S1 and S3 harbored more diverse consortia, while S2 exhibited a markedly simplified composition. S1 was dominated by *Parabacteroides distasonis* (60.87%) and contained a moderate abundance of *Bifidobacterium breve* (1.93%). In contrast, S2 was overwhelmingly dominated by *Escherichia coli* (67.90%), whereas S3 exhibited a high abundance of *B. breve* (40.43%) and a minor proportion of *Bifidobacterium longum* (1.68%) (Figure 1C; Supplementary Table S1).

To isolate *Bifidobacterium* strains, we serially diluted fecal sample S3, plated it on selective media, and incubated it anaerobically. From a single plate, 40 colonies were randomly selected and subjected to full-length 16S rRNA gene PCR and sequencing for species identification. Among these, 22 were identified as *B. breve*, 14 as *E. coli*, and four as *Cutibacterium avidum*. The isolate composition closely mirrored the species-level metagenomic data (Figure 1C; Supplementary Figure S1B), affirming the consistency between culture-dependent and -independent methods. Although *B. longum* was detected at low abundance in S3 by metagenomic analysis (Figure 1C; Supplementary Figure S1B), it could not be recovered under the applied culturing conditions. Similarly, repeated attempts to isolate *Bifidobacterium* from S1 and S2 failed to yield any species beyond *B. breve*, consistent with their low or undetectable abundance in metagenomic profiles (Figure 1C; Supplementary Figure S1B).

A novel *Bifidobacterium* strain was successfully isolated from sample S3 and designated *B. breve* GZX43. Its complete genome was sequenced using Oxford Nanopore technology (Figure 1D). The circular chromosome spans 2,275,544 base pairs with a GC content of 58.89%, in line with other sequenced *B. breve* strains (Bottacini et al., 2014). Genome annotation identified 1,941 CDSs, 53 tRNA genes, nine rRNA genes, and 13 non-coding RNA genes. Of the predicted CDSs, 1,566 (80.7%) were functionally classified via eggNOG, an extension of the COG database. Consistent with previous reports (Bottacini et al., 2014), carbohydrate transport and metabolism (11.65%) and amino acid transport and metabolism (9.81%) were the most enriched functional categories (Supplementary Figure S1C).

### 3.2 Quantifying transformation efficiency in *B. breve* GZX43

**Figure 2:**
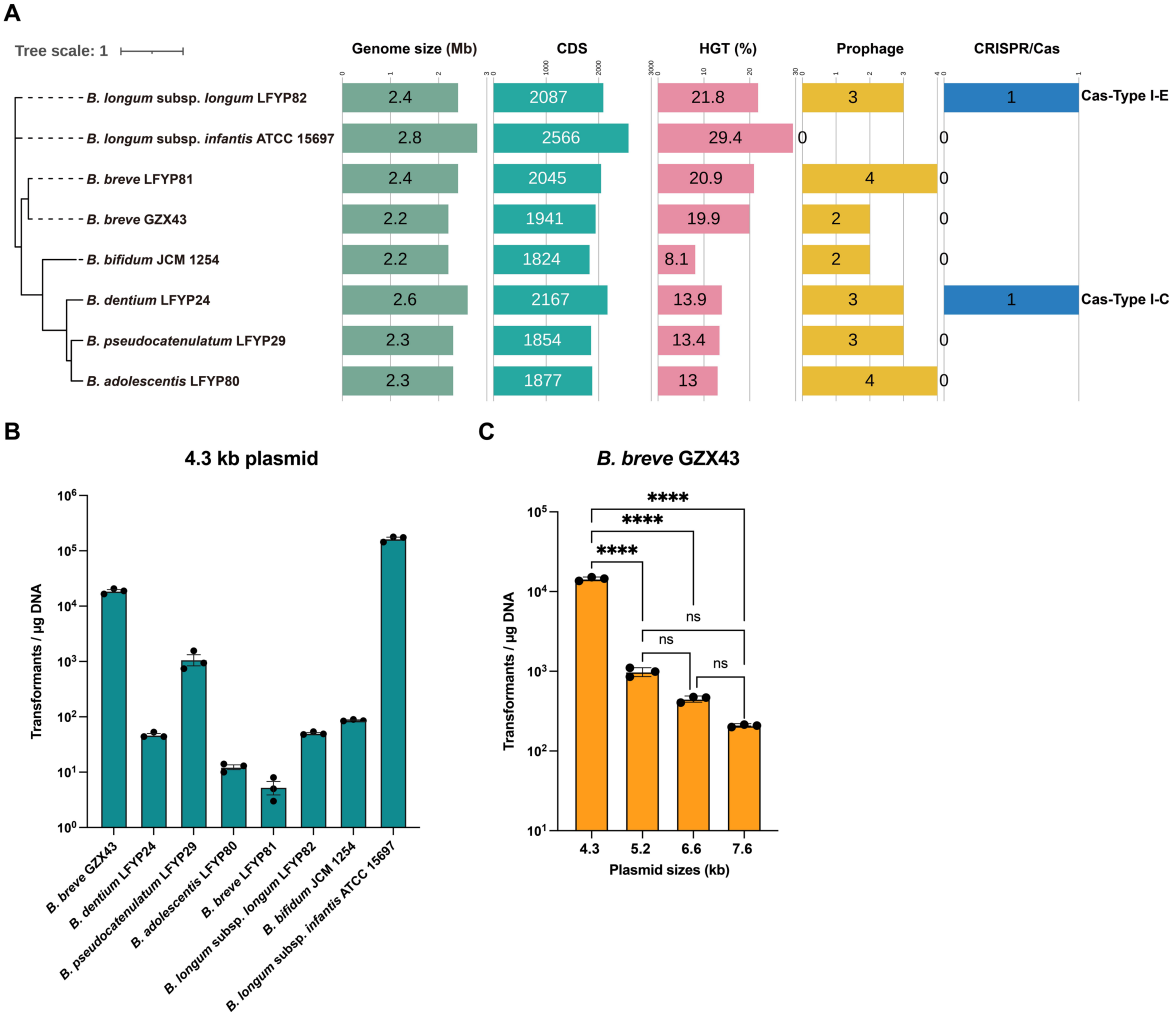
Evaluation of genetic manipulation potential in *Bifidobacterium* strains. **(A)** Phylogenomic comparison of eight *Bifidobacterium* strains: six clinical isolates—one from a Chinese infant (*B. breve* GZX43) and five from a US infant (*B. dentium* LFYP24, *B. pseudocatenulatum* LFYP29, *B. adolescentis* LFYP80, *B. breve* LFYP81, *B. longum* subsp. *longum* LFYP82)—alongside two reference strains (*B. bifidum* JCM 1254 and *B. longum* subsp. *infantis* ATCC 15697). Genomic features compared include genome size, number of protein-coding sequences (CDSs), horizontal gene transfer (HGT) elements, prophage content, and endogenous CRISPR-Cas systems. **(B)** Electroporation-based transformation efficiency of the eight strains using a 4.3-kb plasmid. Data are presented as dot plots showing the mean ± SEM from three biological replicates. **(C)** Effect of plasmid size (4.3–7.6 kb) on transformation efficiency (CFU/μg DNA) in *B. breve* GZX43. Data are shown as dot plots with the mean ± SEM (*n* = 3). Statistical analysis was conducted using one-way ANOVA followed by Tukey’s multiple comparison test. Significance levels are indicated as: not significant (ns) for *p* > 0.05, ****p < 0.0001.

To evaluate the genetic manipulability of *B. breve* GZX43, we conducted a comparative genomic analysis with seven additional *Bifidobacterium* strains preserved in our laboratory: *B. dentium* LFYP24, *B. pseudocatenulatum* LFYP29, *B. adolescentis* LFYP80, *B. breve* LFYP81, and *B. longum* subsp. *longum* LFYP82, all isolated initially from healthy U. S. infants (Feng et al., 2020), along with two reference strains with established genetic systems: *B. bifidum* JCM 1254 and *B. longum* subsp. *infantis* ATCC 15697. Phylogenetic and genomic comparisons produced genome sizes from 2.2 to 2.8 Mb, CDS counts from 1,824 to 2,566, HGT frequencies from 8.1 to 29.4%, and prophage counts varying from 0 to 4 per genome (Figure 2A). Although *B. breve* GZX43 was phylogenetically close to the conspecific *B. breve* LFYP81, it showed marked differences in genome size, number of CDSs, and prophage content, suggesting substantial genetic divergence between Chinese and Western isolates.

Interestingly, only *B. dentium* LFYP24 and *B. longum* subsp. *longum* LFYP82 harbored complete endogenous Type I CRISPR-Cas systems, whereas the remaining strains—including *B. breve* GZX43—lacked this feature (Figure 2A). The absence of CRISPR-Cas machinery in these strains may limit the applicability of CRISPR-based genetic tools.

Due to the abundance and diversity of restriction-modification (R-M) systems in *Bifidobacterium* (Sun et al., 2012; O’Connell Motherway et al., 2014; O’Callaghan et al., 2015), transformation efficiencies are generally low, with a median of ~10^3^ CFU/μg plasmid DNA achieved by electroporation (Fukiya et al., 2012). This poses a major bottleneck for genetic engineering. To assess transformability, we introduced a 4.3 kb shuttle plasmid (pElectro-4.3 kb) into all eight strains via electroporation. The plasmid contained a *Bifidobacterium*-specific replication origin, an *E. coli* replicon, and a spectinomycin resistance gene, and was derived from pDP870 (Klijn et al., 2006). Remarkably, *B. breve* GZX43 exhibited a high transformation efficiency of 1.87 × 10^4^ ± 1,186 CFU/μg DNA—substantially exceeding that of other clinical isolates (Figure 2B). Only the reference strain *B. longum* subsp. *infantis* ATCC 15697 showed higher transformation efficiency, consistent with previous findings that it lacks an R-M system (Tarracchini et al., 2021). Unexpectedly, *B. breve* LFYP81—closely related to GZX43—had the lowest transformation efficiency among all tested strains, highlighting significant strain-dependent variability (Figure 2B). These results confirm that *B. breve* GZX43 is intrinsically amenable to high-efficiency transformation, underscoring its potential as a genetically tractable clinical isolate.

To assess the influence of plasmid size on transformation efficiency, we introduced four plasmids of varying lengths—pElectro-4.3 kb (4.3 kb), pElectro-5.2 kb (5.2 kb), pElectro-6.6 kb (6.6 kb), and pElectro-7.6 kb (7.6 kb)—with identical replication origins into *B. breve* GZX43. A clear inverse relationship emerged: transformation efficiency declined significantly with increasing plasmid size (Figure 2C). When plasmid size reached 7.6 kb, transformation efficiency fell to levels comparable to those of other clinical strains (Figure 2C), indicating that plasmid size constitutes a critical barrier to efficient transformation and may limit the feasibility of certain genetic manipulations, such as gene knockouts, in this strain.

### 3.3 Suicide vector knockout efficiency in *B. breve* GZX43

The clinical isolate *B. breve* GZX43 exhibited a transformation efficiency second only to the model strain *B. longum* subsp. *infantis* ATCC 15697 (Figure 2B). The latter has been widely reported as a successful recipient for suicide vector-based gene knockouts via either single-crossover plasmid insertion or double-crossover allelic exchange (Sakanaka et al., 2019; Higgins and Ryan, 2021; Hirano et al., 2021; Arzamasov et al., 2022). The high transformation efficiency observed in *B. breve* GZX43 suggested its potential suitability for genetic manipulation using similar approaches.

To evaluate the feasibility of double-crossover allelic exchange in *B. breve* GZX43, we constructed a 3.2 kb suicide plasmid containing a spectinomycin resistance cassette flanked by ~500 bp homologous arms. This plasmid was introduced into *B. breve* GZX43 via electroporation, and transformants were selected on spectinomycin-supplemented media (Supplementary Figure S2A). Two target genes with distinct sizes and functions were chosen for this study: GE000081 (L-lactate dehydrogenase, 951 bp) and GE001410 (Type II restriction enzyme, 1,296 bp). These plasmids were named pSUC43-ΔGE000081 and pSUC43-ΔGE001410, and their transformations yielded 19 and 22 colonies, respectively. However, PCR screening revealed that all transformants retained the wild-type (WT) genotype, with no detectable single-crossover (SCO) or double-crossover (DCO) events (Supplementary Figures S2B,C), indicating a recombination frequency of zero (Supplementary Figure S2D). Repeated experiments produced identical results (Supplementary Figure S2D), demonstrating that suicide vector-mediated allelic exchange is ineffective for targeted mutagenesis in *B. breve* GZX43.

### 3.4 Shuttle vector-driven deletion of *pyrE* in *B. breve* GZX43

Given the failure of suicide vector-mediated recombination, we explored whether a shuttle vector system, less reliant on high transformation efficiency, could enable genome editing in *B. breve* GZX43. As a proof of concept, we targeted *pyrE*, a gene commonly used in counterselection systems, to assess the feasibility of rapid and efficient markerless gene deletion via double-crossover recombination.

**Figure 3:**
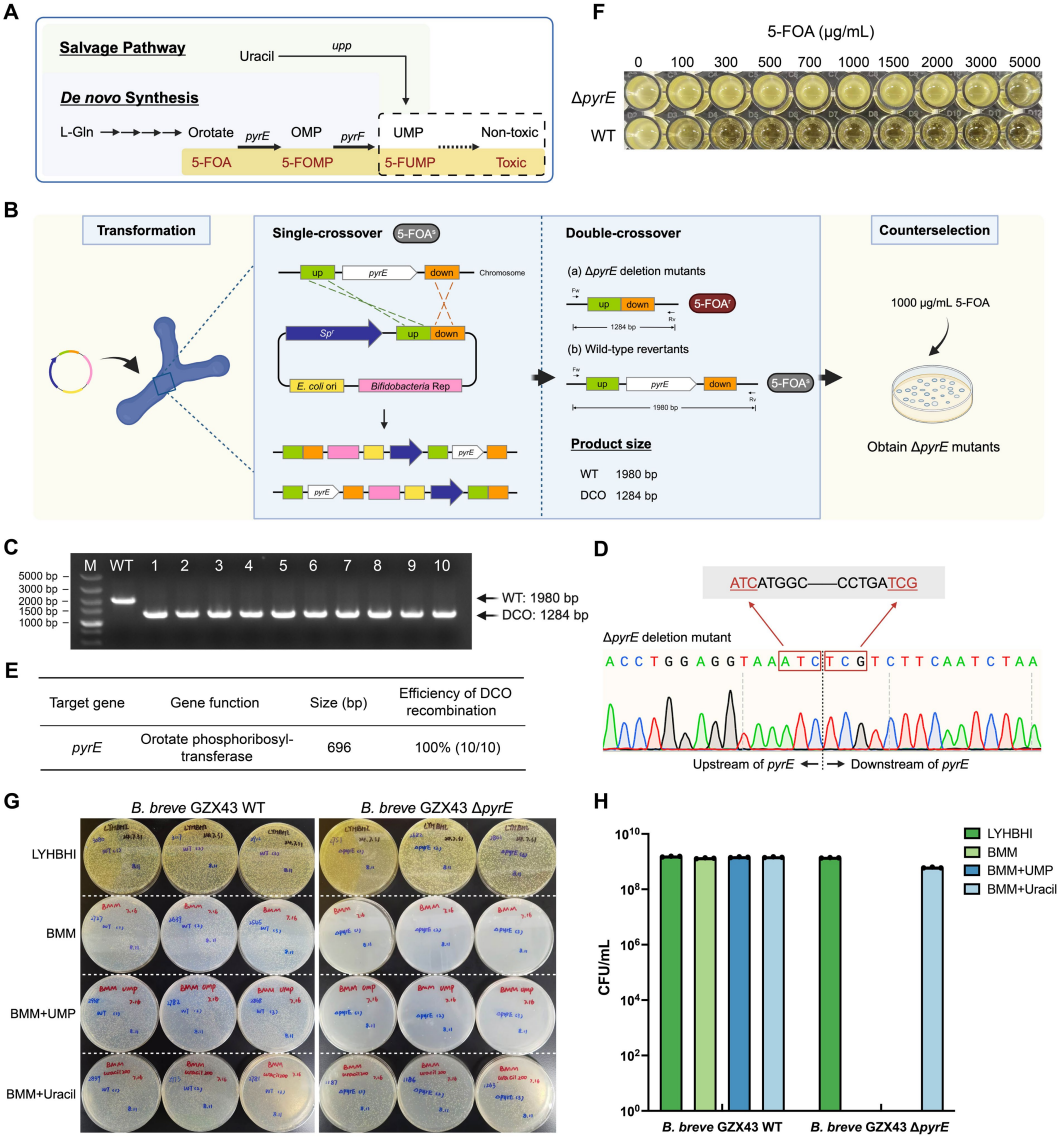
Targeted deletion of *pyrE* in *B. breve* GZX43 via homologous recombination using a shuttle vector. **(A)** Pyrimidine biosynthesis pathway in *Bifidobacterium*. The pyrimidine analog 5-fluoroorotic acid (5-FOA) is converted by *pyrE*-encoded orotate phosphoribosyltransferase into 5-fluorouridine 5′-monophosphate (5-FUMP), which inhibits DNA synthesis and incorporates into RNA, leading to cell death. Disruption of *pyrE* abrogates *de novo* pyrimidine synthesis, rendering cells dependent on exogenous uracil via the salvage pathway. **(B)** Schematic of the *pyrE* gene knockout procedure. A shuttle vector with 500 bp homologous arms was transformed into *B. breve* GZX43. Following one round of non-selective subculturing to facilitate double-crossover (DCO) recombination, colonies were selected on plates containing 1,000 μg/mL 5-FOA. **(C)** PCR screening of ten randomly selected colonies from 5-FOA plates using locus-specific primers (Fw: *pyrE* left-F, Rv: *pyrE* right-R). M: DNA marker; WT: wild-type control; Lanes 1–10: Δ*pyrE* candidates; DCO: double-crossover mutants. **(D)** Sanger sequencing confirmation of *pyrE* deletion in DCO mutants. **(E)** Efficiency of shuttle vector-mediated homologous recombination at the *pyrE* locus. **(F)** 5-FOA resistance assay for WT and Δ*pyrE* strains across a concentration range of 0–5,000 μg/mL. **(G,H)** Nutritional phenotype analysis. WT and Δ*pyrE* strains were grown on LYHBHI (rich medium), BMM (minimal medium), BMM supplemented with 1.8 mM uridine 5′-monophosphate (UMP), and BMM supplemented with 1.8 mM uracil. Panel G shows qualitative assessment on agar plates; Panel H shows quantitative CFU counts. Data are presented as dot plots with the mean ± SEM of three biological replicates.

Pyrimidine biosynthesis pathways, due to their evolutionary conservation, have served as robust counterselection tools in diverse organisms, including bacteria (Yang et al., 2021; Wu et al., 2022) (e.g., *Streptomyces rimosus*, *Acinetobacter baumannii*), yeast (Hirashima et al., 2006), and archaea (Liu et al., 2011) (e.g., *Haloferax mediterranei*, *Haloarcula hispanica*). In the *de novo* pyrimidine biosynthesis pathway of *Bifidobacterium* (Sakaguchi et al., 2013), orotate is converted to orotidine 5′-monophosphate (OMP) by orotate phosphoribosyltransferase (*pyrE*), and then to uridine 5′-monophosphate (UMP) by orotidine 5′-monophosphate decarboxylase (*pyrF*). Both steps are essential for cell survival. However, in the presence of 5-fluoroorotic acid (5-FOA), a structural analog of orotate, *pyrE* converts it into 5-fluoroorotidine 5′-monophosphate (5-FOMP), which is subsequently metabolized by *pyrF* into the toxic compound 5-fluorouridine 5′-monophosphate (5-FUMP), disrupting RNA processing and DNA synthesis, and ultimately causing cell death (Figure 3A). Consequently, cells lacking *pyrE* or *pyrF* exhibit resistance to 5-FOA. In *B. longum* 105-A, *pyrE* has been validated as a bidirectional selection marker (Sakaguchi et al., 2013): Δ*pyrE* mutants are 5-FOA resistant and uracil auxotrophs, failing to grow in minimal medium lacking uracil.

To construct Δ*pyrE* mutants, we engineered the shuttle vector pKO43-Δ*pyrE*, which includes ~500 bp flanking sequences homologous to the *pyrE* locus. Following transformation into *B. breve* GZX43, a single subculture was performed in antibiotic-free LYHBHI to facilitate recombination, after which the culture was plated on 5-FOA-containing medium (1,000 μg/mL) to select for Δ*pyrE* mutants (Figure 3B). Of the 269 colonies obtained, 10 were randomly selected for verification. PCR analysis using primers *pyrE* left-F and *pyrE* right-R (Supplementary Table S3) yielded the expected 1,980 bp amplicon for the WT strain and 1,284 bp for Δ*pyrE* mutants (Figure 3C). Sanger sequencing confirmed the precise deletion of the 696 bp *pyrE* coding region (Figure 3D). The combination of shuttle vector-mediated recombination and 5-FOA counterselection achieved a 100% deletion efficiency among screened clones (Figure 3E), demonstrating the utility of *pyrE* as a robust counterselection marker in *B. breve* GZX43.

Then, we performed phenotypic characterization of the Δ*pyrE* strains. In a resistance assay using media containing 0–5,000 μg/mL 5-FOA, we observed that the WT strain was unable to grow when the 5-FOA concentration reached 1,000 μg/mL, while the mutant strain was still able to grow at a concentration as high as 5,000 μg/mL (Figure 3F; Table 2). Previous studies have reported that the strain *B. longum* 105-A Δ*pyrE* cannot grow in bifidobacterial minimal medium (BMM) lacking uracil (Sakaguchi et al., 2013). We replicated this by plating *B. breve* GZX43 WT and Δ*pyrE* strains on BMM with or without uracil or UMP supplementation (Figures 3G,H). Both strains grew comparably on rich medium (LYHBHI), but Δ*pyrE* mutants failed to grow on unsupplemented BMM. Supplementation with 1.8 mM uracil fully restored growth, whereas equivalent concentrations of UMP were unable to do so, likely due to the poor membrane permeability of phosphorylated nucleotides. These results confirmed the successful markerless deletion of *pyrE* in *B. breve* GZX43 and validated the expected uracil auxotrophy phenotype.

**Table 2:** Differential 5-FOA resistance profiles between Δ*pyrE* and WT strains.

5-FOA μg/mL/OD_600_	0	100	300	500	700	1,000	1,500	2,000	3,000	5,000
*B. breve* GZX43 Δ*pyrE*	1.712	1.636	1.624	1.506	1.430	1.392	1.296	1.248	1.086	0.700
*B. breve* GZX43 WT	1.682	0.661	0.576	0.321	0.160	0.097	0.049	0.027	0.016	0.002

### 3.5 Dual-plasmid knockout enables deletion of non-selectable genes

Although the deletion of *pyrE* in *B. breve* GZX43 was highly efficient, this success depended on strong counterselection via 5-FOA. To develop a more universally applicable system for gene knockout, particularly for genes without selectable phenotypes, we designed a novel dual-plasmid strategy to enable rapid and scarless genome editing.

**Figure 4:**
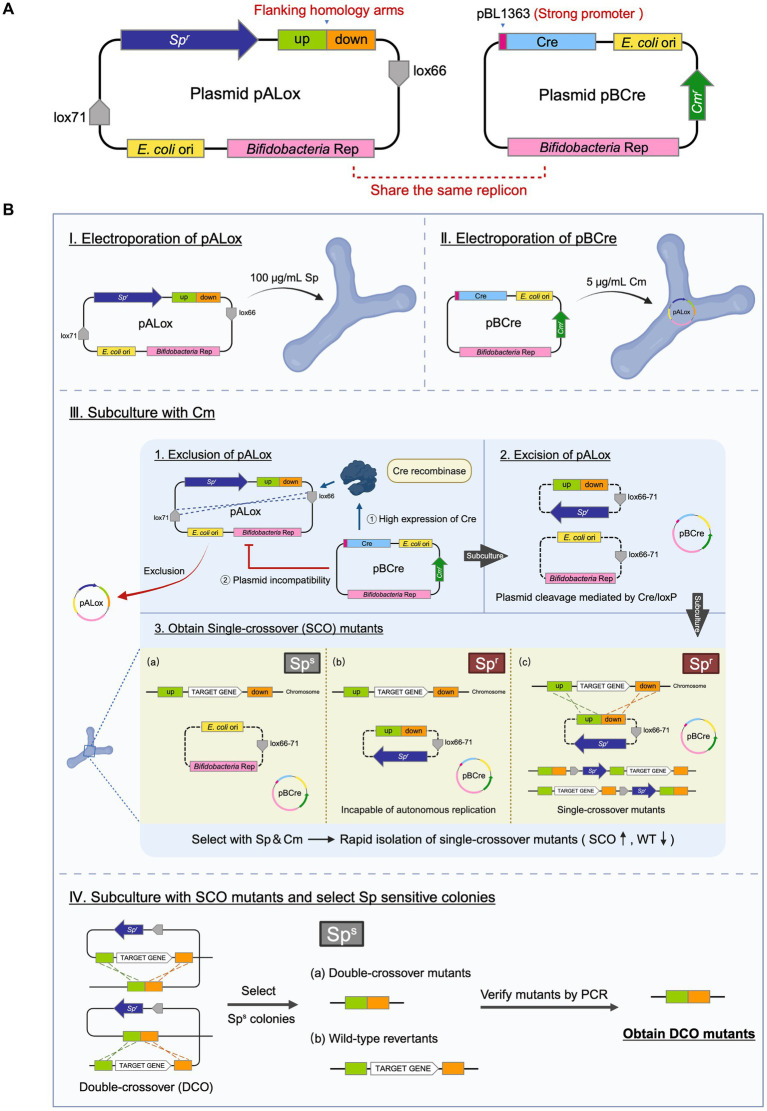
Dual-plasmid strategy for targeted gene knockout in *B. breve* GZX43. **(A)** Schematic of the dual-plasmid system. The knockout plasmid pALox carries 500 bp homology arms and a spectinomycin resistance (Sp^r^) marker flanked by two directly oriented loxP sites; the bifidobacterial replicon lies outside this excisable cassette. The helper plasmid pBCre carries the same replicon to ensure incompatibility with pALox and expresses Cre recombinase under a strong promoter, enabling excision of the loxP-flanked region. **(B)** Workflow of dual-plasmid-mediated gene knockout. First, pALox is electroporated into *B. breve* GZX43, and transformants are selected on spectinomycin (Sp). These are made competent and subsequently transformed with pBCre, followed by selection on chloramphenicol (Cm). PCR confirms dual-plasmid presence. Serial subcultures in Cm alone eliminate intact pALox through: (1) plasmid incompatibility, which disrupts pALox replication, and (2) Cre-mediated excision of the Sp^r^-homology cassette from pALox. Only recombinants with chromosomally integrated Sp^r^ are retained under dual antibiotic pressure, enriching for single-crossover (SCO) events. SCO strains are subcultured in Cm without Sp to promote the second recombination, yielding either wild-type revertants or double-crossover (DCO) mutants, which are confirmed via PCR.

The inducible plasmid self-destruction (IPSD) approach, depending on the Cre-loxP system, has previously been employed in *Lactobacilli* and certain *B. longum* strains to enable SCO recombinant selection (Zuo et al., 2019; Zuo and Marcotte, 2021). However, its efficiency relies on the activity of the L-arabinose-inducible *araC*-P*_BAD_* promoter, which has weak activity in our clinical *Bifidobacterium* isolates. Even at L-arabinose concentrations of 0.5, 1, and 2%, no significant Cre recombinase expression was obtained (data not shown). To circumvent this limitation, we developed a two-plasmid system comprising pALox and pBCre (Figure 4A).

The pALox plasmid contains ~500 bp homologous arms flanking the target gene and a spectinomycin resistance (Sp^r^) marker, all enclosed between directly oriented lox66 and lox71 sites. The replicons for *E. coli* and *Bifidobacterium* lie outside the excisable cassette. The second plasmid, pBCre, expresses Cre recombinase under the control of the strong *B. longum* subsp. *longum* NCC2705-derived promoter pBL1363 (Klijn et al., 2006). Additionally, pBCre shares the same replicon as pALox, causing plasmid incompatibility within the host. This facilitates the clearance of unrecombined but replication-competent pALox plasmids, potentially persisting due to the incomplete efficiency of Cre-mediated recombination.

The workflow for this dual-plasmid system begins with the electroporation of pALox into *B. breve* GZX43, followed by selection on spectinomycin (Sp) (Figure 4B, Step I). Next, the Sp^r^-positive clones were made electrocompetent and transformed with pBCre, with subsequent selection on chloramphenicol (Cm) (Step II). PCR validated the presence of both plasmids, after which the strains were serially subcultured using Cm as the sole antibiotic. The strong promoter pBL1363 on pBCre drove high-level expression of Cre recombinase, which catalyzed the excision of the Sp^r^–homology arm cassette flanked by lox66/lox71 sites from the replicable portion of pALox. As the two plasmids share a common replicon and cannot stably coexist, plasmid incompatibility, combined with Cre-mediated excision, eliminates intact, non-integrated pALox, thereby minimizing false positives during Sp-based selection. Dual selection with Sp and Cm enriched for SCO integrants (Step III). Finally, Sp selection was withdrawn while Cm selection was maintained to promote the second homologous recombination event. This step yielded either WT revertants or desired DCO mutants, which were identified by PCR screening (Step IV).

### 3.6 Targeted inactivation of GE001229 using a dual-plasmid knockout system

**Figure 5:**
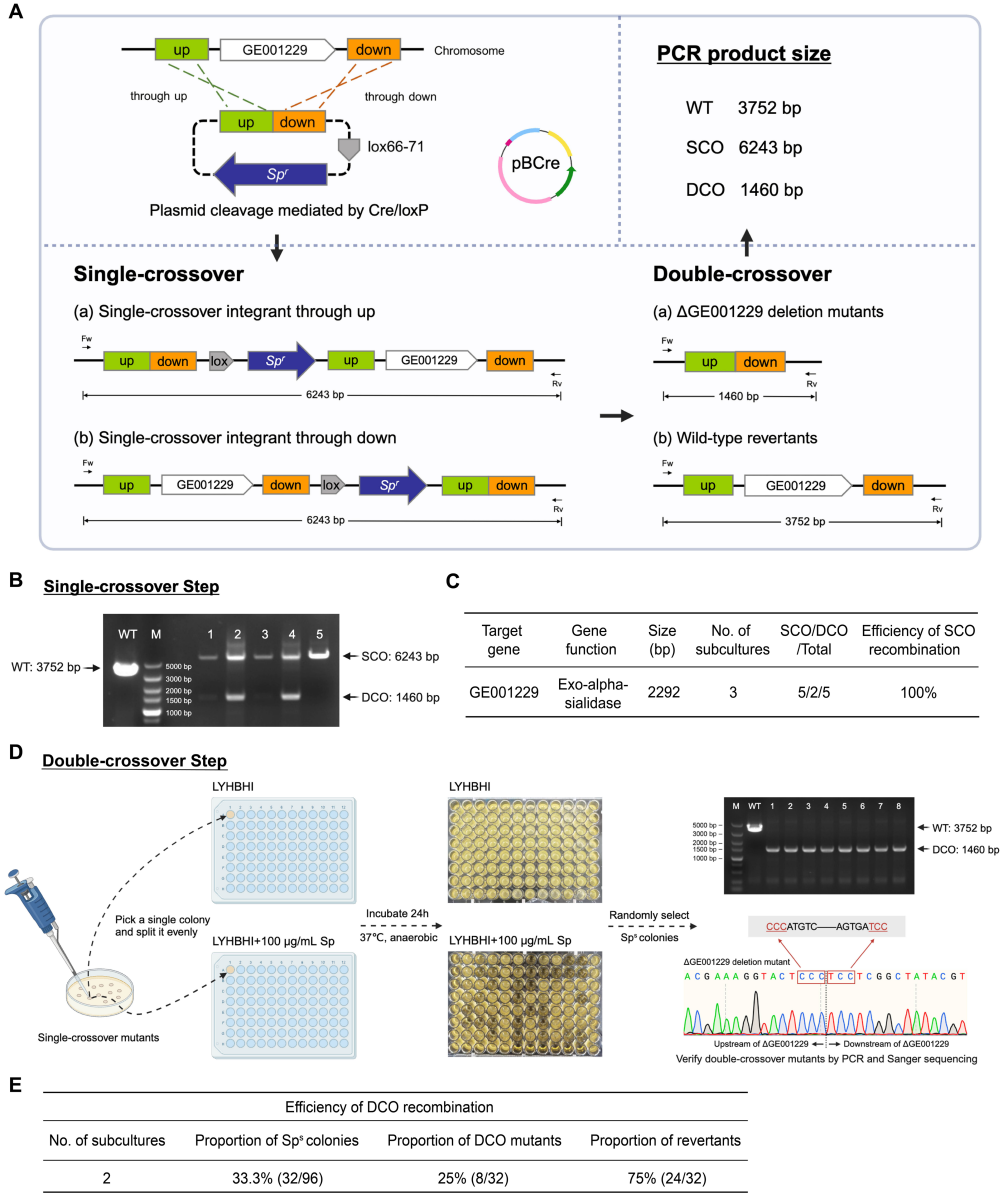
Targeted deletion of GE001229 in *B. breve* GZX43 using a dual-plasmid knockout system. **(A)** Schematic of the GE001229 deletion strategy. *B. breve* GZX43 was sequentially transformed with pALox-ΔGE001229 and pBCre. Following transformation, cells were subcultured three times in LYHBHI medium supplemented with chloramphenicol (Cm) to facilitate clearance of intact pALox-ΔGE001229. Transformants were plated on LYHBHI agar containing both spectinomycin (Sp) and Cm to select exclusively for recombination events. PCR analysis of amplicon sizes using locus-specific primers distinguished single-crossover (SCO), double-crossover (DCO), and WT genotypes. **(B)** Identification of SCO mutants. Five colonies randomly picked from plates containing Cm and Sp were analyzed by PCR using primers flanking the GE001229 locus (Fw: GE001229 left-F, Rv: GE001229 right-R). M: DNA marker; WT: wild-type control; Lanes 1–5: candidate *B. breve* GZX43 ΔGE001229 SCO mutants. **(C)** SCO recombination efficiency at the GE001229 locus in *B. breve* GZX43 mediated by the dual-plasmid system. **(D)** DCO mutant isolation and verification. Purified SCO clones were subcultured twice in Sp-free LYHBHI medium to promote a second homologous recombination event. Cultures were then plated on antibiotic-free LYHBHI agar to isolate single colonies. Each colony was inoculated in parallel into (1) LYHBHI with Sp and (2) antibiotic-free LYHBHI. After 24 h of anaerobic incubation, colonies that grew only in the antibiotic-free medium were selected as putative DCO mutants. These candidates were further validated by PCR using GE001229-specific primer pairs (Fw: GE001229 left-F, Rv: GE001229 right-R), with successful DCO events yielding a 1,460 bp amplicon. Final confirmation was performed via Sanger sequencing to ensure precise gene deletion. **(E)** DCO recombination efficiency at the GE001229 locus in *B. breve* GZX43 using the dual-plasmid knockout system.

To assess the efficacy of the dual-plasmid gene knockout system, we targeted GE001229, a non-selectable gene in *B. breve* GZX43. This gene encodes exo-*α*-sialidase, an enzyme essential for bifidobacterial degradation of sialylated human milk oligosaccharides (HMOs) and mucin-derived glycans (Kiyohara et al., 2011; Nishiyama et al., 2017). The knockout plasmid pALox-ΔGE001229 was constructed and introduced into *B. breve* GZX43 according to the workflow previously described (Figure 5A).

After three consecutive subcultures in Cm-supplemented LYHBHI broth, transformants were plated on LYHBHI agar containing both Sp and Cm to select for SCO recombinants. PCR screening of five randomly chosen colonies confirmed that all carried the expected 6,243 bp SCO amplicon (Figure 5B), indicating 100% SCO recombination efficiency at this locus (Figure 5C). Notably, two of the five colonies also exhibited a 1,460 bp DCO product, suggesting an unexpectedly high frequency of homologous recombination in *B. breve* GZX43. These findings underscore the dual-plasmid system as a robust and efficient approach for generating SCO mutants.

To isolate DCO mutants (Figure 5D), a verified SCO colony was subcultured twice in Sp-free LYHBHI broth to facilitate the second homologous recombination event. As DCO mutants are expected to lose Sp resistance, cultures were plated on non-selective agar to obtain single colonies, which were subsequently replica-inoculated into both Sp-containing and antibiotic-free LYHBHI media. After 24 h of anaerobic incubation, 32 out of 96 colonies (33.3%) exhibited Sp sensitivity. PCR and Sanger sequencing using primers GE001229 left-F and GE001229 right-R (Supplementary Table S3) confirmed that only 8 of these Sp-sensitive colonies carried the expected 1,460 bp DCO product, while the remaining 24 represented WT revertants (Figure 5E).

Next, we evaluated the growth of the ΔGE001229 DCO mutants on representative sialylated substrates commonly found in the infant gut, including sialylated HMOs (e.g., 3′-SL and 6′-SL). Mutants exhibited growth profiles comparable to the WT strain (data not shown), thereby precluding direct phenotypic validation via substrate utilization. However, the ΔGE001229 mutants consistently formed smaller and thinner colonies and displayed significantly slower growth in liquid culture, requiring approximately 2–3 times longer to reach the same OD_600_ as the WT. This phenotype may reflect the broader role of exo-α-sialidase in carbohydrate metabolism and growth regulation.

In addition, we assessed the presence of the two plasmids in both SCO and DCO mutants. PCR analysis of four SCO mutants confirmed the complete loss of the intact plasmid pALox, with only the integrated Sp^r^-homology cassette yielding a detectable PCR product (Supplementary Figures S4A,B), highlighting the high efficiency of loss of pAlox in this system. In contrast, plasmid pBCre was retained in both SCO and DCO mutants under continuous Cm selection, but was progressively lost upon serial passaging under non-selective conditions (Supplementary Figures S4B–D). PCR analysis further confirmed the complete loss of plasmid pBCre in all eight Cm-sensitive ΔGE001229 DCO single colonies isolated from the fourth subculture. These results demonstrated that plasmid pBCre could be efficiently cured, facilitating iterative scarless genome editing in *B. breve* GZX43.

Collectively, these results demonstrate that the dual-plasmid strategy enables efficient, scarless gene deletion in *B. breve* GZX43, offering a valuable tool for genetic manipulation in this species.

## 4 Discussion

In this study, we isolated a highly abundant clinical strain of *Bifidobacterium breve*, designated GZX43, from a healthy Chinese infant. We sequenced its complete genome, finding that it exhibited an exceptionally high transformation efficiency, which substantially surpassed previously characterized clinical isolates. To evaluate its genetic tractability, we examined two classical double-crossover gene knockout strategies. While a suicide vector-based allelic exchange approach was unsuccessful, we generated a *pyrE* mutant using a shuttle vector using the counterselectable phenotype conferred by 5-fluoroorotic acid (5-FOA) sensitivity. For efficient and flexible gene deletion in this clinically relevant strain, we developed a dual-plasmid system that significantly enhanced the selection efficiency of the first homologous recombination step, enabling scarless, high-efficiency gene knockouts. Our work demonstrates that this dual-plasmid system enables gene deletion in a previously intractable clinical *B. breve* strain, underscoring the need to tailor genetic tools to the unique physiology and genomic features of bifidobacterial isolates.

For genetic engineering and functional studies in *Bifidobacterium*, efficient transformation is critical, as it directly impacts the success of gene editing and other plasmid-based molecular applications. In our experiments, transformation efficiencies varied markedly across eight *Bifidobacterium* strains during electroporation. This variability may stem from multiple factors, including R-M systems, oxygen sensitivity, and differences in cell wall structure (Park et al., 2019), while replicon compatibility and plasmid stability may influence plasmid maintenance after transformation. Among these factors, R-M systems are widely recognized as the primary genetic barrier to transformation. However, accurately predicting active R-M systems remains challenging due to their strain-specific complexity—including considerable diversity in both type and abundance across *Bifidobacterium* strains, as well as the confounding influence of host-derived methylation patterns on plasmid propagation. High-resolution techniques such as single-molecule real-time (SMRT) sequencing are often required to precisely identify R-M loci and their recognition motifs (O’Connell Motherway et al., 2014). Therefore, systematic characterization of R-M systems is essential for improving transformation efficiency and advancing the development of genetic tools in *Bifidobacterium*.

Although CRISPR-Cas systems are widely used in gut microbiome research and genetic engineering (Zheng et al., 2024), their application in *Bifidobacterium*—particularly in clinical isolates—remains limited, regardless of whether exogenous or endogenous systems are employed. To date, exogenous CRISPR-Cas9 has been successfully applied only in *Bifidobacterium animalis* AR668 (Li et al., 2023). However, the large size of Cas9-encoding plasmids requires host strains with high transformation efficiency, and constitutive Cas9 expression often induces cytotoxicity, complicating their use (Misra et al., 2019). Endogenous CRISPR-Cas systems have also been explored for gene editing in *Bifidobacterium* (Pan et al., 2022; Han et al., 2024), but their utility is constrained by two key limitations. First, the presence of *cas* genes is highly strain-specific—not all strains possess complete CRISPR-Cas systems. For example, our bioinformatic analysis of eight *Bifidobacterium* strains revealed only two with complete Type I systems, greatly limiting the broader applicability of this method. Second, although Type I CRISPR-Cas systems predominate in *Bifidobacterium*, they are inherently more difficult to engineer than the more commonly used Type II systems (Li et al., 2023). Recent innovations, such as the CRISPR-Cas9 cytosine base editor system (cBEST), have enabled efficient single-base edits in several *Bifidobacterium* strains (Pan et al., 2022; Lin et al., 2025). However, this method remains restricted to C-to-T conversions and cannot mediate large deletions or diverse base modifications. Additional challenges include strain-dependent variation in editing efficiency due to epigenetic factors, reduced transformation efficiency due to R-M systems, and off-target effects.

Because *B. breve* GZX43 lacks an endogenous CRISPR-Cas system and the suicide vector-based allelic exchange strategy failed, these two classical editing approaches are not viable for this strain. The transformation efficiency of *B. breve* GZX43 may still fall below the levels required for successful homologous recombination using non-replicative plasmids. Previous studies indicate that a transformation efficiency exceeding 10^5^ CFU/μg DNA is needed to obtain recombinants using non-replicative plasmids, given the low integration frequency (10^−3^–10^−5^ ipc) (Sakaguchi et al., 2012). For instance, in *B. longum* subsp. *infantis* ATCC 15697, whose intrinsic transformation efficiency is higher than that of GZX43, transformation efficiency was significantly improved by using plasmid artificial modification (PAM) to bypass R-M systems, which enabled the successful construction of mutants through suicide vector-based allelic exchange (Higgins and Ryan, 2021). A similar strategy may be necessary for *B. breve* GZX43, though it is technically demanding and operationally complex.

The two-step, double-crossover, markerless gene deletion strategy remains one of the most practical approaches for editing clinical isolates. When shuttle plasmids are used, this approach bypasses the limitations imposed by low transformation efficiency. However, replicative vectors relying on antibiotic selection often yield high false-positive rates, necessitating the development of enhanced strategies to improve selection for the desired mutants. Systems such as IPSD (Zuo et al., 2019) and Ts plasmids (Sakaguchi et al., 2012; Kozakai et al., 2024) have been developed to facilitate the initial homologous recombination step and reduce the effort required to isolate SCO mutants. Yet, in our evaluation across multiple strains (data not shown), both IPSD and the Ts plasmid pKO403 failed to effectively eliminate intact vectors, leading to a persistently high rate of WT escape clones.

To overcome these limitations, we developed a novel knockout strategy tailored to *B. breve* GZX43. This dual-plasmid system utilizes plasmid pALox for gene deletion and pBCre to eliminate intact pALox. Unlike the IPSD method, our system does not rely on L-arabinose-inducible promoters, which often vary in activity across species. Instead, pBCre constitutively expresses Cre recombinase under the strong pBL1363 promoter, known to function robustly in both *B. longum* (Klijn et al., 2006) and *B. breve* strains. The plasmid backbone contains a pDP870-derived replicon, enabling stable replication in a wide range of *Bifidobacterium* species (Klijn et al., 2006; Grimm et al., 2014; Osswald et al., 2015), and was successfully maintained across all eight tested strains in our lab. These results suggest that our dual-plasmid system is not only efficient in *B. breve* GZX43, but may also have the potential to apply across diverse *Bifidobacterium* species.

The dual-plasmid system also has its own limitations. The inherent incompatibility between the two plasmids may pose conceivable challenges in strains with particularly low transformation efficiencies, where co-transformation is less likely to occur. Furthermore, the main limitation of this system is its inability to substantially increase the frequency of DCO events. This likely stems from the absence of the RecBCD recombination pathway in most *Bifidobacterium* species (Schell et al., 2002; Guglielmetti et al., 2013), which results in low spontaneous recombination rates without selection pressure. Interestingly, during the targeted deletion of GE001229, we observed that 40% (2/5) of SCO mutants spontaneously resolved into DCO mutants without subculturing. Furthermore, after serial subculturing of SCO mutants, the DCO recombination efficiency reached 33.3% (32/96), although most were WT revertants, and only 25% were true DCO mutants. These results suggest that *B. breve* GZX43 may possess inherently high recombination activity.

To improve the efficiency of DCO mutant selection, several strategies have been developed. One method involves introducing an incompatible plasmid that encodes the *repA* gene, which promotes the excision of integrated vectors during the second recombination event through plasmid incompatibility (Hirayama et al., 2012). Although the initial plasmid lacks *repA*, it shares the same origin of replication, and the resulting plasmid competition drives recombination-based resolution. Another widely used strategy involves the use of counterselectable markers to enrich for DCO recombinants. To date, *pyrE* remains the only counterselectable marker successfully applied in *Bifidobacterium* (Sakaguchi et al., 2013). In other bacterial systems, several alternative counterselection approaches have been explored. The *sacB* gene from *Bacillus subtilis*, which encodes levansucrase, generates toxic levan in the presence of sucrose and is effective in many Gram-negative bacteria (Gay et al., 1985; Qin et al., 2021; Zhou et al., 2025); however, its use in *Bifidobacterium* is limited due to low expression levels, enzyme instability, host intolerance to levan, and endogenous sucrose metabolism, which together lead to frequent escape of non-recombinant cells. Other systems include the mutant *pheS* gene (pheS*), encoding a phenylalanyl-tRNA synthetase *α* subunit hypersensitive to p-chlorophenylalanine (p-Cl-Phe), which has proven effective in various bacteria (Miyazaki, 2015; Kino et al., 2016; Zhou et al., 2017), and the *upp* gene, encoding uracil phosphoribosyltransferase, which sensitizes cells to 5-fluorouracil (5-FU) and has been employed in *Clostridium* (Boudignon et al., 2023), *Lactobacillus* (Goh et al., 2009), and *Pseudomonas* (Wang et al., 2015). Toxic proteins, such as MazF and HicA, which trigger cell death through RNA cleavage or translation inhibition, also offer strong counterselection potential (Zhang et al., 2006; Gc et al., 2023). Still, their utility in *Bifidobacterium* remains constrained by the lack of universal, tightly regulated inducible promoters. Broader application of these systems in *Bifidobacterium* will require deeper understanding of host-specific metabolic features, toxin sensitivities, and promoter compatibility. In this study, we generated a *pyrE* deletion mutant in *B. breve* GZX43—the second such report in *Bifidobacterium*, following *B. longum* 105-A (Sakaguchi et al., 2013). Plasmid curing via serial subculturing restored spectinomycin (Sp) sensitivity, enabling the reuse of Sp-selectable vectors in the Δ*pyrE* background. We propose engineering future knockout plasmids by inserting a highly expressed *pyrE* gene between the homology arms and the Sp resistance cassette in the pALox backbone. Gene deletions would then be conducted in the uracil auxotrophic Δ*pyrE* background, with 5-FOA used to counterselect DCO mutants, improving selection efficiency at both SCO and DCO stages.

Overall, this work presents a robust and adaptable framework for the genetic engineering of clinically isolated *Bifidobacterium* strains—a task often hindered by limited knowledge of their unique genetic backgrounds. Such knowledge gaps pose significant challenges and typically necessitate prolonged, iterative optimization of genetic tools. For example, the extensively studied model strain *B. breve* UCC2003, after more than two decades of research, became the first *Bifidobacterium* strain for which a high-density, randomly barcoded transposon mutant library was generated, enabling genome-scale insights into host–microbe interactions and colonization determinants (Shiver et al., 2025). For future efforts targeting genetic manipulation of other clinical isolates, a comprehensive evaluation of strain-specific features, including the presence of CRISPR-Cas systems, R-M systems, transformation efficiency, and recombination capacity may be necessary. Based on these assessments, appropriate counterselectable markers should be selected to ensure compatibility with the host’s physiology. Although engineering newly isolated clinical strains remains challenging, adopting rational, tailored strategies will advance functional genomics in *Bifidobacterium* and other host-associated bacteria.

## Data Availability

The raw metagenomic and whole-genome sequencing data generated in this study have been deposited in the NCBI public repository (https://www.ncbi.nlm.nih.gov/), under BioProject accession number PRJNA1277733.

## Footnotes

^1^ https://www.snapgene.com/

^2^ https://www.graphpad.com/
