# Supplementary material for: Scarless gene disruption enabled by a dual-plasmid knockout platform in a clinical infant-derived Bifidobacterium breve strain
Source: Front Microbiol. 2025 Sep 10;16:1653505. doi: 10.3389/fmicb.2025.1653505 (PMC12459721; doi:10.3389/fmicb.2025.1653505)
Supplement: Supplementary file 1 [file Data_Sheet_1.docx]

***Supplementary Figures***

**Scarless gene disruption enabled by a dual-plasmid knockout platform in a clinical infant-derived *Bifidobacterium breve* strain**

Zhenxuan Gao, Lihui Feng^*^

Institutes of Biomedical Sciences, Fudan University, Shanghai, China

^*^ Correspondence to: Institutes of Biomedical Sciences, Fudan University, Shanghai, China.

E-mail: lihuifeng@fudan.edu.cn.

**

**

**Figure S1. Gut microbiota composition of the three infant fecal samples.**

(A) A radial phylogenetic tree illustrates taxonomic relationships from phylum (innermost ring) to species (outermost ring). A peripheral heatmap depicts species-level relative abundances across the three samples (S1, S2, and S3), using a gradient scale (red: higher group-average abundance; blue: lower).

(B) Heatmap showing Z-score-normalized species-level abundance of the top 30 taxa across S1, S2, and S3 (red: high abundance; blue: low).

(C) Functional classification of genes annotated by eggNOG. Functional categories are listed on the x-axis, while the y-axis shows the absolute gene numbers and their relative abundance percentages.

**

Figure S2. Targeted gene deletion in *B. breve* GZX43 via suicide vector-mediated homologous recombination.**

(A) Schematic of gene knockout via double-crossover (DCO) allelic exchange. The suicide vector containing a spectinomycin resistance (Sp^r^) gene flanked by 500 bp homology arms matching the 5′ and 3′ ends of the target gene was developed and introduced into *B. breve* GZX43. Through two homologous recombination events, the Sp^r^ cassette was inserted into the genome or replaced the target gene, allowing for the selection of single-crossover (SCO) and DCO mutants on Sp-containing media.

(B) Nineteen colonies from Sp-containing plates were subjected to PCR using GE000081-specific primers (Fw: GE000081 left-F, Rv: GE000081 right-R). M: DNA marker; WT: wild-type control; Lanes 1–19: ΔGE000081 candidate mutants.

(C) Twenty-two colonies from Sp-containing plates were screened via PCR using GE001410*-*specific primers (Fw: GE001410 left-F, Rv: GE001410 right-R). M: DNA marker; WT: wild-type control; Lanes 1–22: ΔGE001410 candidate mutants.

(D) Recombination efficiency of the suicide vector-mediated gene knockout approach in B. breve GZX43.


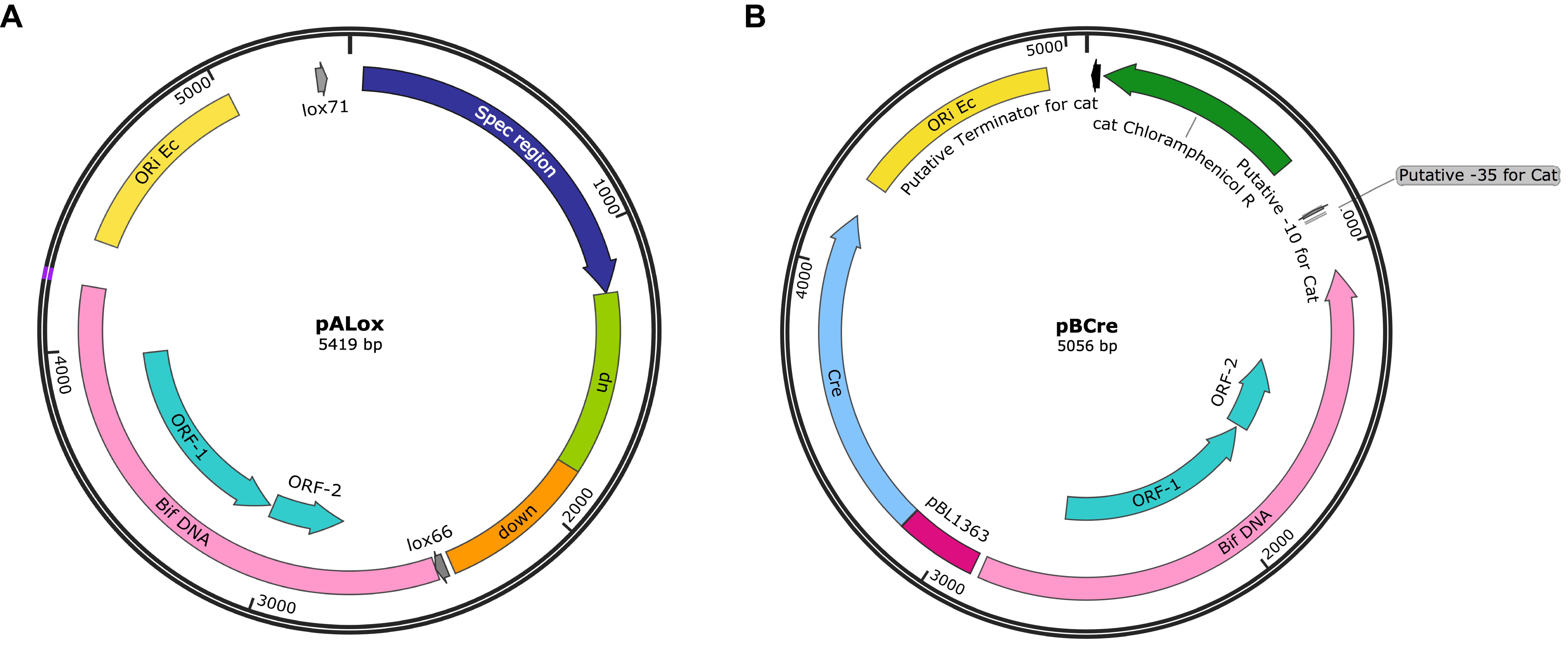


**Figure S3. Physical maps of plasmids pALox and pBCre (related to Figure 4).**

(A) pALox: includes the bifidobacterial replicon (Bif DNA) derived from pDP870, an E. coli replicon (ORi Ec), and a spectinomycin resistance gene (Spec) flanked by two directly oriented loxP sites (lox66 and lox71). The 500 bp upstream (top) and downstream (bottom) homology arms flank the target gene region.

(B) pBCre: contains the same bifidobacterial and E. coli replicons as pALox, along with the Cre recombinase gene under the control of the strong pBL1363 promoter and a chloramphenicol resistance gene (*cat*). Plasmid maps were generated using SnapGene v4.1.8 software.





**Figure S4. PCR-based identification of the loss of pALox and pBCre.**

(A) Schematic diagram illustrating the primers and corresponding target products used for the identification of pALox and pBCre. Primers spanning the lox71 site (Fw: pALoxCheck-1-F, Rv: pALoxCheck-1-R) were used to detect the presence of the intact pALox. Primers targeting the spectinomycin resistance gene (Fw: pALoxCheck-2-F, Rv: pALoxCheck-2-R) were employed to assess whether the excised spectinomycin-containing fragment of pALox had been integrated into the genome. The presence of pBCre was confirmed using primers (Fw: pBCre-Scr-F, Rv: pBCre-Scr-R).

(B) Four single-crossover (SCO) colonies were subjected to PCR using three pairs of primers to detect the presence of pALox and pBCre. M: DNA marker; pALox: plasmid pALox-ΔGE001229; pBCre: plasmid pBCre; WT: wild-type control; Lanes 1-4 (SCO): ΔGE001229 SCO mutants.

(C) Loss of pBCre during successive subcultures. Single colonies were picked from the second to fourth generation subcultures, and patched on non-selective LYHBHI plates and LYHBHI plates containing Cm5 (chloramphenicol 5µg/mL).

(D) Eight single colonies from the fourth subculture of double-crossover (DCO) mutants, along with liquid cultures from generations 1 to 4, were tested for the presence of pBCre via PCR (Fw: pBCre-Scr-F, Rv: pBCre-Scr-R). M: DNA marker; pBCre: plasmid pBCre; WT-1 and WT-2: wild-type controls; Lanes sub 1–4: liquid cultures from the first to fourth subcultures; Lanes 1–8: single colonies of ΔGE001229 DCO mutants from the fourth subculture that are sensitive to Cm5.
